# U. S. Dispensatory

**Published:** 1877-10-20

**Authors:** 


U. S. DISPENSATORY.—The Profession will be pleased to learn that a new edition of the U. S. Dispensatory is announced. Dr. Geo. B. Wood, Dr. H. C. Wood, and Prof. Bridges are the authors.

